# t-plasminogen activator inhibitor-1 polymorphism in idiopathic pulmonary arterial hypertension

**DOI:** 10.4103/0971-6866.44103

**Published:** 2008

**Authors:** Sujana Katta, Shivani Vadapalli, B. K. S. Sastry, Pratibha Nallari

**Affiliations:** Department of Genetics, University College of Science, Osmania University, Hyderabad, Andhra Pradesh, India; 1 Cardiology Unit, CARE Hospital, The Institute of Medical Sciences, Hyderabad, Andhra Pradesh, India

**Keywords:** Fibrinolytic pathway, idiopathic pulmonary arterial hypertension, plasminogen activator inhibitor-1, thrombosis, tissue remodelingIntroduction

## Abstract

**AIM:**

The aim of the present study was to identify the possible genotypic association of 3’UTR *Hind III* polymorphism of Plasminogen activator Inhibitor-1 (PAI-1) gene with idiopathic pulmonary arterial hypertension (IPAH).

**BACKGROUND:**

IPAH is a disorder with abnormally raised mean pulmonary arterial pressure and increase in the resistance to blood flow in pulmonary artery. One of the pathological features seen is development of intraluminal thrombin deposition leading to thrombosis. Plasminogen activator inhibitor-1 is an important inhibitor of the fibrinolytic system; its up-regulation may suppress fibrinolysis and result in an increased risk of thrombosis.

**METHOD:**

Blood samples from 54 IPAH patients and 100 healthy voluntary donors were analyzed by PCR-RFLP method for 3’UTR *Hind III* polymorphism.

**RESULTS AND DISSCUSSION:**

A significant association of Hd2 allele with the disease was observed. Raised mean level of right ventricular systolic pressure was observed in the Hd2/Hd2 genotypic patients, strengthening the role of Hd2 allele in the disease progression. Our data suggests an association of Hd2/Hd2 genotype, which may lead to the up-regulation of PAI-1 gene leading to increased levels of PAI-1, which is seen in IPAH. PAI-1 competes with plasminogen activators and hinders the normal mechanism of plasminogen activation system and leads to thrombosis and formation of plexiform lesions in the lung tissue, further strengthening its role in tissue remodeling and disease progression.

## Introduction

Idiopathic pulmonary arterial hypertension (IPAH) is a disorder characterized by an abnormally raised mean pulmonary arterial pressure greater than 25 mm Hg at rest or greater than 30 mm Hg during exercise. Due to changes in pulmonary arterial pressure, an increase in the resistance to blood flow through arteries is observed. Important pathological feature of IPAH is the widespread development of *in situ* thrombosis of the small pulmonary arteries with intraluminal thrombin deposition.[1] Abnormalities in platelet activation, function; and biochemical features of a procoagulant environment within the pulmonary vasculature support a role of thrombosis in disease initiation.[2] Interactions between growth factors, platelets, and the vessel wall suggest that thrombin may play a pivotal role in many of the pathobiological processes and disease progression as described for IPAH.

Plasminogen activator inhibitor-1 (PAI-1) is a 50-kDa glycoprotein, encoded by PAI-1 gene localized to 7q21.3-q2. It is a major regulator of plasminogen activation and is the principal inhibitor of tissue plasminogen activator (tPA) and urokinase (uPA).[3] The activators of plasminogen lead to intravascular fibrinolysis, the physiological breakdown of blood clots, which catalyzes the conversion of the zymogen plasminogen to plasmin. Fibrin deposition and lysis must be balanced to maintain and remold the hemostatic seal during repair of an injured vessel wall. In inflammatory conditions, fibrin is deposited in tissues and PAI-1 appears to play a significant role in the progression to fibrosis. Lower PAI levels may lead to suppression of fibrinolysis and, on the contrary, a more rapid degradation of the fibrin. Plasminogen activator inhibitor-1 is an important inhibitor of the fibrinolytic system and elevated levels may suppress fibrinolysis, resulting in an increased risk of thrombosis.

Plasminogen activator inhibitor-1 is synthesized by a variety of cell types like endothelial cells, hepatocytes, and platelets, and its expression is regulated by growth factors and hormones.[4] PAI-1 synthesis by endothelial cells can be stimulated by a number of inflammatory mediators, including endotoxin, interleukin-1, and tumor necrosis factor; as well as fibroblast growth factor-2, angiotensin-2, and lipids. Plasminogen activator inhibitor-1 is secreted in active form, but subsequent conformational changes can make it inactive.

The genetic variation in 3’UTR in the gene sequence of PAI has a *Hind III* restriction site and appears to play an important regulatory role in eventual antigen expression.[5] Furthermore, specific external factors may also selectively enhance the regulation of each allelic form of the gene.[6]

The 3’UTR polymorphisms of the PAI-1 gene have been found to contribute in the regulation of its expression, which suggests a genotype-specific interaction between these polymorphisms.[7] The RFLP/HindIII polymorphism in the 3’ region of the fibrinolytic protein plasminogen activator inhibitor (PAI-1) influences the plasma levels of PAI-1 and is reported to be associated with the development of cardiovascular disease.[8] Polymorphisms in the PAI-1 promoter region (4G/5G) and 3’UTR were found to be associated with venous thrombosis.[9] Hence genotyping of PAI-1 was carried out to identify if any specific genotype was associated with IPAH.

## Materials and Methods

Blood samples from 54 confirmed IPAH patients (29 females, 25 males) were collected from Care Hospitals, Hyderabad, A. P.; and 100 control blood samples (5 mL) from Gandhi Hospitals, Hyderabad; informed consent of the all the subjects and approval of the institutional ethics committee were obtained for the genotyping of 3’UTR of PAI-1 gene.

DNA isolation was carried out by nonenzymatic salting out procedure.[11] PCR was performed for genomic DNA to amplify the gene 3’UTR (untranslated region) of PAI-1 gene.

A 12.5-µL PCR mixture was prepared containing 100 ng of genomic DNA, 0.2 mM of both forward and reverse primers, 10 mM dNTP's, 10XPCR buffer [10 mM Tris-HCl (pH 8.3), 50 mM KCl, 2 mM MgCl_2_], 2.5% of formamide, 2U of Taq DNA polymerase (Sigma Aldrich, Germany). Primers sequences used: forward primer: 5’ AGCAATCCACCTGTCTCGGC 3’, reverse primer: 5’ TCCTGACCTCAGGTGATCCG 3’.[12] The PCR conditions optimized for the amplification of PAI-1 were for 40 cycles with denaturation at 94°C for 30 s; annealing temperature at 60°C for 30 s; and elongation at 72°C for 1 min with initial denaturation of 95°C for 5 min and final extension of 72°C for 5 min.

PCR-amplified products were digested by adding 5 U of 1X Hind III restriction enzyme (Bangalore Genei, India) with an overnight incubation at 37°C. The products after digestion were electrophoresed on 2% agarose gel (Sigma Aldrich, Germany), at 150 V, containing 1 µL/10 mL ethidium bromide, and the genotypes were observed by visualizing under UV light. The genotypes were determined by the presence of allele Hd1, identified by presence of 2 bands — a 292 bp and a 102 bp — as a result of restriction digestion; and allele Hd2, by the presence of a 394-bp undigested product which results from a G→C substitution at nt 14,699 that abolishes the Hind III restriction site.

The allele and genotypic frequencies were computed to test for the deviation from Hardy-Weinberg equilibrium using chi-square test. The odds test of association was performed for the different genotypes at 95% confidence intervals.

## Results

The frequency of Hd1/Hd1, Hd1/Hd2, and Hd2/Hd2 genotypes was found to be 22%, 62%, and 16% in the control subjects and 20.37%, 33.33%, and 46.29% in IPAH respectively [Table 1], indicating a predominance of Hd1/Hd1, Hd1/Hd2 genotype in the control subjects and Hd2/Hd2 genotype in the disease group, with the difference being statistically significant (χ^2^ 17.67; *P*<.05;.01). The allelic frequency of Hd1 and Hd2 was found to be 0.53 and 0.47 in control group and 0.37 and 0.62 in IPAH, with no deviation from Hardy-Weinberg expectations either in the control or IPAH, signifying random allelic distribution in the local population.

**Table 1:** Frequency distribution and allelic and genotypic distribution of plasminogen activator inhibitor-1 genotypes in controls and idiopathic pulmonary arterial hypertension

Genotypes	Genotype frequencies								Alleles	Allelic frequencies	
			
	Control				IPAH					Control	IPAH
					
	Obs (n)	Exp	χ^2^	n %	Obs (n)	Exp	χ^2^	n %			
Hd1/Hd1	22	28.09	1.32	22	11	7.39	1.76	20.37	Hd1	0.53	0.37
Hd1/Hd2	62	49.82	2.97	62	18	24.77	1.85	33.33			
Hd2/Hd2	16	22.09	1.67	16	25	20.75	0.87	46.29	Hd2	0.47	0.62
χ^2^			5.97				4.48				

*P <0.05; **P <0.01

The Odds ratio for Hd2/Hd2 genotype was found to be significantly associated with the disease. [Hd2/Hd2 Vs Hd1/Hd1 (OR-3.12, 1.08 - 9.17), Hd2/Hd2 Vs Hd1/Hd2 (OR- 5.32, 2.20 -13.31)] as presented in Table 2. A significant association of Hd2 allele with IPAH was observed in comparison to Hd1 allele (OR 1.91, 1.15-3.18).

**Table 2:** Odds ratio of Plasminogen activator Inhibitor 1 genotypes in IPAH compared to the control group

PAI-1 Genotypes	Odds ratio	95% CI
Hd1 Vs Hd2	0.52	0.31-0.86
Hd2 Vs Hd1	1.91*	1.15-3.18*
Hd1/Hd1 Vs Hd2/Hd2	0.32	0.10 - 0.92
Hd1/Hd1 Vs Hd1/Hd2	1.72	0.64 – 4.60
Hd2/Hd2 Vs Hd1/Hd1	3.12*	1.08 - 9.17*
Hd2/Hd2 Vs Hd1/Hd2	5.32*	2.20 - 13.31*
Hd1/Hd2 Vs Hd1/Hd1	0.58	0.21 - 1.55

* *P*<0.05

The frequency distribution of PAI-1 genotypes with respect to gender is shown in Table 3. Inter-group comparison based on gender shows predominance of Hd2/Hd2 genotype in male control subjects (26%) as compared to males in IPAH cases (14.81%). Among women the frequency of Hd1/Hd2 and Hd2/Hd2 genotypes was found to be 25.92% and 18.25% respectively in IPAH cases, much higher than the 17% and 8% found in control subjects. An intra-group analysis revealed a significant association of Hd2/Hd2 with respect to gender (χ^2^ 5.33; *P* < 0.05), with predominance of Hd2 allele in female patients.

**Table 3:** Frequency distribution of plasminogen activator inhibitor-1 genotypes in IPAH with respect to gender

Gender	Genotypes					
	
	Hd1/Hd1		Hd1/Hd2		Hd2/Hd2	
Male	15	11.11	22	20.37	26	14.81
Female	12	9.25	17	25.92	8	18.51
χ^2^	0.03		0.93		5.33*	

* *P* < 0.05

Hemodynamic parameters are influenced by the disease progression with progressive rise in pulmonary vascular resistance. Increase in the right ventricular systolic pressures [equivalent to the pulmonary artery systolic pressures (sPAP)] is associated with right ventricular failure, which can be obtained by calculating the right ventricular pressure gradient and right atrial pressure (RAP) gradient.

The mean levels of RVSP with respect to PAI-1 genotypes were (X±SD) 101.90 ± 24.84 for Hd1/Hd1, 100.52 ± 30.79 for Hd1/Hd2, and 102.29 ± 16.23 for Hd2/Hd2, with mean RVSP being higher in the Hd2/Hd2 genotypes, further supporting the Hd2 association with IPAH; and the association can be correlated to the severity of the disease, increased mPAP pressures, and Tricuspid regurgitation (TR) velocity [Table 4].

**Table 4:** Mean levels of right ventricular systolic pressure with respect to genotypes of PAI-1

PAI-1 Genotypes	RVSP(mmHg) X SD n
Hd1/Hd1	101.90 24.84 11
Hd1/Hd2	100.52 30.79 25
Hd2/Hd2	102.29 16.23 17

## Discussion

Synthesis and expression of PAI-1 at transcriptional level are, to a large extent, stimulated by transforming growth factor β-1 (TGF β-1), released from the alpha granules of platelets after vascular injury.[13] In the present study, an association of Hd2/Hd2 genotype with IPAH was observed, supporting earlier studies. This can be explained on the basis of interaction of TGF-β and coagulants pathway that may be involved in the pathogenesis of IPAH.

An interesting observation of the study was the predominance of Hd2 allele in women that was found to be significantly associated with the disease. Since female preponderance is reported in IPAH,[14] it is possible that this allele could be one of the predisposing risk factors in women, acting in conjuncture with other influencing factors such as sex hormones. Our hypothesis is supported by a previous study where significantly increased t-PA antigen and t-PA activity in correlation with mPAP, as well as increased PAI-1 activity, was reported in women with IPAH, and this presence of gender differences in the plasmin- and thrombin-activation system in IPAH leading to an antifibrinolytic/prothrombotic state was suggested as a possible explanation for the female predominance in the disease.[15]

The odds ratio of Hd2 allele was found to be significant when compared to HD1 allele in IPAH. (OR, 1.91; CI, 1.15 to 3.18). This observation further supports the earlier reports that have implicated the Hd2 allele for the increased levels of PAI-1. Elevated PAI-1 levels impair the fibrinolytic pathway by competing with tissue plasminogen activator (t-PA), thereby inhibiting plasminogen activating system. Thrombosis of pulmonary vessels is a common finding in IPAH, where it is found with a frequency of 30% to 60%.[16] These thrombotic events are likely to be involved in the progression of the disease by hindering tissue repair and thus contributing to the altered vasculature/architecture that is commonly seen in IPAH.

With respect to clinical parameters, an increase in the mean level of right ventricular systolic pressure was observed in the Hd2/Hd2 genotypic patients, strengthening the role of Hd2 allele in the etiopathogenesis of IPAH in correlation to severity and poor prognosis.

## Conclusion

Our data suggests an association of Hd2/Hd2 genotype with the disease, and may be responsible for up-regulation of PAI-1 which competes with plasminogen activators and impairing the normal mechanism of plasminogen activation system. This activation may contribute significantly to IPAH by promoting thrombosis and fibrosis, leading to tissue remodeling of the lung. The Hd2 allele could be a predisposing factor for female preponderance in IPAH, but this needs to be ascertained on a larger sample size.
